# Serological evaluation of selected vector-borne pathogens in owned dogs from northern Spain based on a multicenter study using a commercial test

**DOI:** 10.1186/s13071-020-04172-5

**Published:** 2020-06-10

**Authors:** David Díaz-Regañón, Xavier Roura, María L. Suárez, Marta León, Ángel Sainz

**Affiliations:** 1 Department of Animal Medicine and Surgery, College of Veterinary Medicine, Complutense University of Madrid, Avda. Puerta de Hierro s/n, 28040 Madrid, Spain; 2 Hospital Clínic Veterinari, Universitat Autònoma de Barcelona, Carrer de lʼHospital s/n, 08193 Bellaterra Barcelona, Spain; 3 Department of Veterinary Clinical Sciences, Rof Codina Veterinary Teaching Hospital, Faculty of Veterinary Medicine, University of Santiago de Compostela, 22702 Lugo, Spain; 4 Boehringer Ingelheim Animal Health Spain S.A, Sant Cugat del Vallés, Spain

**Keywords:** Leishmaniasis, Ehrlichiosis, Anaplasmosis, Borreliosis, Dirofilariosis, Prevalence, Southern Europe

## Abstract

**Background:**

Environmental conditions in northern Spain allow the development of different arthropods involved in the transmission of significant canine vector-borne pathogens. The aim of the study was to systematically assess seroprevalence rates for *Leishmania infantum*, *Ehrlichia canis*, *Anaplasma* spp., *Dirofilaria immitis* and *Borrelia burgdorferi*, and risk factors in dogs from all regions of the north of Spain.

**Methods:**

A total of 556 dogs were included in this study between January 2017 and December 2018, belonging to 30 practices covering all regions in northern Spain (Galicia, Asturias, Cantabria, Basque Country, Navarra, Aragon and Catalonia). All practices were located in the north of every region. Blood samples were analyzed using the 4DX SNAP® test (IDEXX Laboratories, Westbrook, Maine, USA) for the detection of *D. immitis* antigen and *E. canis*, *B. burgdorferi* and *Anaplasma* spp. antibodies. *Leishmania* SNAP*®* test (IDEXX Laboratories) was used for detection of *L. infantum* antibodies. Associations between prevalence of canine vector-borne pathogens, epidemiological and clinical signs data were statistically analyzed.

**Results:**

The overall prevalence rates were 8.99% for *L. infantum*, 1.26% for *Anaplasma* spp., 0.9% for *E. canis*, 0.72% for *B. burgdorferi*, and 0.18% for *D. immitis*. Globally, 11.33% of the dogs included in the study were positive to any tested vector-borne pathogen. *Leishmania infantum* seroprevalence was the highest and the only one detected in all the regions. *Leishmania infantum* seropositivity was associated with age > 10 years-old, outdoor access, anemia, fever, dermatological signs, lympadenomegaly, muscular atrophy, ocular signs and renal disease. *Ehrlichia canis* seropositivity was associated with the summer season and living in urban areas. Apathy, weakness, anorexia, weight loss, anemia, fever and gastrointestinal clinical signs were also associated with *E. canis* antibody detection. Living in a rural area was also a risk factor for *Anaplasma* spp. and *B. burgdorferi* seropositivity.

**Conclusions:**

To our knowledge, this is the first multicenter survey performed in northern Spain assessing different canine vector-borne diseases from all regions. Results show the presence of autochthonous cases of these diseases. The vector-borne pathogens found in this study should be included in the differential diagnosis in dogs from some areas previously considered non-endemic for these pathogens.
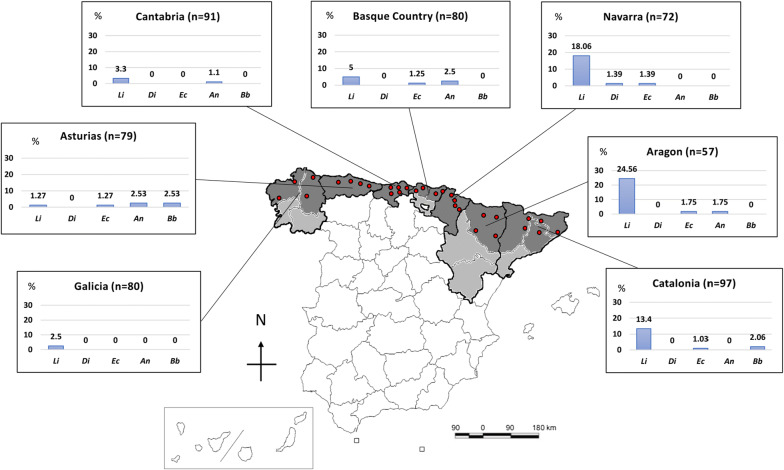

## Background

Canine vector-borne pathogens are a range of microorganisms transmitted by ectoparasites mainly ticks, fleas, mosquitoes and sand flies. The distribution ranges of these agents are increasing worldwide and their epidemiology seems to be constantly evolving due to factors such as climate change, movement of companion animals travelling with their owners, the development of outdoor activities, and environmental changes including the creation of recreational parks with forest fragmentation and the potential increased contact with vectors and sylvatic reservoirs, among others [1, 2].

The north of Spain has been traditionally considered a non-endemic area for some of the vector-borne pathogens usually found in the rest of the country, for example *Leishmania infantum*, mainly due the specific climate conditions of this area [3, 4]. The north and northwest of Spain constitute one of the biogeographical regions of the country, with a humid Atlantic climate, with mild winters and summers [3, 5]. The regions near the Pyrenees (Navarra, north of Aragon, and northwest of Catalonia) have a Continental climate, with cold winters and mild summers [6]. The northeast of the country is included in the Mediterranean biogeographical region, being also wet due to the influence of the Mediterranean Sea, with mild winters and hot summers [5, 6].

Thus, climatic properties found in northern Spain allow the detection of different arthropod vectors frequently affecting dogs, such as *Ixodes* spp., *Dermacentor* spp., *Rhipicephalus sanguineus* and *Phlebotomus perniciosus* [6, 7]. Some of the pathogens potentially transmitted by these vectors are remarkable not only from the animal health point of view but also within the framework of human health. Canine vector-borne diseases include parasitic diseases such as babesiosis, dirofilariosis and leishmaniasis, and bacterial diseases such as anaplasmosis, borreliosis and ehrlichiosis.

Leishmaniasis caused by *L. infantum* is a zoonotic disease with the dog as the main reservoir in Spain, where it is transmitted by phlebotomine sand flies of the genus *Phlebotomus*. Canine leishmaniasis constitutes a systemic chronic disease characterized by several clinical presentations from subclinical to severe and fatal disease [4]. Clinical signs include lymphadenopathy, dermatitis, alopecia, uveitis, onychogryphosis, lameness, weight loss, cachexia, epistaxis, anemia and proteinuria, among many others [4, 8]. Previous studies have reported low seroprevalence rates for *L. infantum* in northern Spain (except in some areas of northeast and northwest of the country) [3, 7, 9]. In some autonomous communities (the Basque Country, Navarra or Aragon) seroprevalence studies in dogs are scarce or even absent in owned dogs. Some prevalence studies performed in stray dogs and wild reservoirs [10], human population [11] and sand flies [12] in these areas suggest a potential underestimation of the prevalence in dogs from northern Spain.

*Dirofilaria immitis* is a nematode transmitted by mosquitoes of the genera *Aedes*, *Anopheles* and *Culex*. This parasitic infection causes a disease known as dirofilariosis or heartworm disease because parasitic adult worms live in the right side of the heart and pulmonary arteries [13, 14]. *Dirofilaria immitis* has been previously reported and associated with dogs living in the Mediterranean basin (which provides optimum temperature and humidity to viable mosquito population) but is not endemic in northwestern and north-central areas of Spain [9]. The clinical signs associated with dirofilariosis include exercise intolerance, dry chronic cough, weakness, weight loss, epistaxis, cyanosis and pulmonary edema [15].

Canine monocytic ehrlichiosis is a tick-borne bacterial disease transmitted by *R. sanguineus* with *Ehrlichia canis* as the causative agent [16]. Previous studies have described a wide distribution in the country and high seroprevalence rates of *E. canis* in dogs from some areas of northern Spain [9]. Clinical signs for ehrlichiosis include weakness, lethargy, exercise intolerance, fever, anorexia, weight loss, lymphadenomegaly, splenomegaly, hepatomegaly, diarrhea, vomiting, hemorrhage, epistaxis, uveitis, and respiratory and sometimes neurological signs [17].

The species of *Anaplasma* affecting dogs in Spain are *A. phagocytophilum* (mainly transmitted by *Ixodes ricinus* in Europe), the causative agent of canine granulocytic anaplasmosis, which may produce a zoonotic disease [18, 19], and *A. platys*, the causative agent of thrombocytopenic anaplasmosis and transmitted by *R. sanguineus* [17]. Infection with *Anaplasma* spp. can be asymptomatic or cause some unspecific clinical signs. Clinical signs of granulocytic anaplasmosis are fever, lethargy, anorexia, splenomegaly, and sometimes neurological and orthopedic signs [17]. Thrombocytopenic anaplasmosis affects platelets and clinical signs include fever, lethargy, anorexia, weight loss, pale mucous membranes, petechiae, nasal discharge and lymphadenomegaly [17]. Antibodies against *Anaplasma* spp. have been detected in recent studies throughout the country [9], and *Anaplasma* spp. have also been detected in ticks collected from dogs in some areas of the north [6]. *Anaplasma* spp. infect a wide variety of domestic and wild vertebrate hosts [17].

Finally, the spirochete *Borrelia burgdorferi* also affects a wide variety of hosts including dogs and humans, causing Lyme disease, and is transmitted by *I. ricinus* [20]. Most infected dogs remain without clinical signs and, when presented, are unspecific. Borreliosis has been associated with hyperthermia, anorexia, lameness, lymphadenopathy and glomerulonephritis [20]. Antibodies against *B. burgdorferi* have been reported in wild canids [5] and in owned dogs [21] in some areas of Spain.

To the best of our knowledge, these vector-borne pathogens have never been evaluated in some areas in the north of Spain. Thus, the aims of the study were to systematically determine the seroprevalence of selected vector-borne pathogens (*Leishmania infantum*, *Ehrlichia canis*, *Anaplasma* spp., *Dirofilaria immitis* and *Borrelia burgdorferi*) in dogs with and without suggestive clinical signs of a vector-borne disease, and the assessment of epidemiological variables as a possible risk factor in all regions of northern Spain.

## Methods

### Blood samples and questionnaire with epidemiological data

**Fig. 1:**
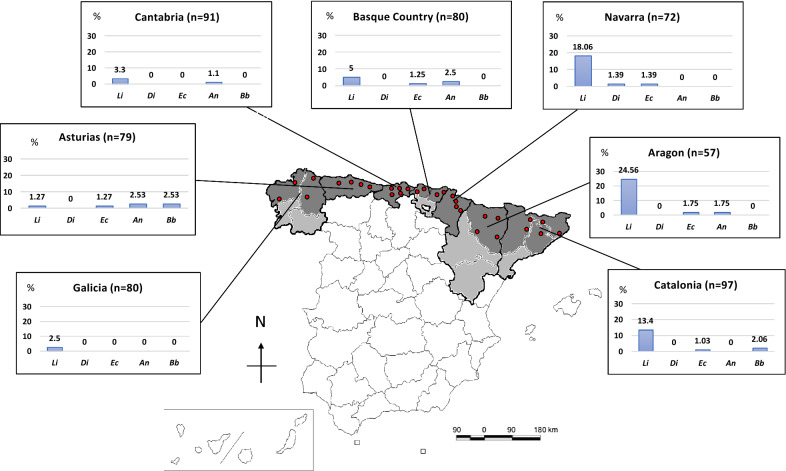
Map with the location of different veterinary practices included in the study and prevalence rates of each canine vector-borne pathogens according to different areas. *Abbreviations*: Li, *Leishmania infantum*; Di, *Dirofilaria immitis*; Ec, *Ehrlichia canis*; An, *Anaplasma* spp.; Bb, *Borrelia burgdorferi*

A multicenter study was performed recruiting a total of 556 dog blood samples from 30 private veterinary practices belonging to seven autonomous communities: Galicia (*n* = 4; 80 dogs); Asturias (*n* = 4; 79 dogs); Cantabria (*n* = 5; 91 dogs); the Basque Country (*n* = 4; 80 dogs); Navarra (*n* = 4; 72 dogs); northern Aragon (*n* = 4; 57 dogs); and northern Catalonia (*n* = 5; 97 dogs). In communities with several provinces, samples were systematically collected from the most northern provinces from every autonomous community. Specifically, samples were collected from A Coruña and Lugo in Galicia, Bizkaia and Gipuzkoa in the Basque Country, Huesca in Aragon and Barcelona, Girona and Lleida in Catalonia (Fig. 1).

Local veterinarians of each area were asked to randomly collect blood samples and complete a questionnaire with clinical and epidemiological data for a maximum of 10 dogs without clinical signs and 10 dogs with clinical signs, suggestive of any vector-borne diseases, including anaplasmosis, borreliosis, dirofilariosis, ehrlichiosis and leishmaniasis.

Clinical signs suggestive for each of these diseases included in the study were defined as follows: fever, anorexia, lethargy, lameness, lymphadenopathy, diarrhea and vomiting, epistaxis, and splenomegaly for anaplasmosis [17]; fever, apathy, weakness, anorexia, weight loss, lymphadenomegaly, splenomegaly, hepatomegaly, epistaxis and neurological signs for ehrlichiosis [17]; weight loss, apathy, exercise intolerance, dyspnea/tachypnea, cough, tachycardia and vena cava syndrome for dirofilariosis [13]; lymphadenomegaly, dermatological signs, ocular signs, weight loss, muscular atrophy, lethargy, anemia, epistaxis, hepatomegaly, splenomegaly, lameness, proteinuria, and fever for leishmaniasis [8]; and lameness, fever, anorexia and proteinuria for borreliosis [20].

Additionally, epidemiological data were recorded from each dog regarding: date of the sample collection; signalment; tick infestation at the time of the physical exam; use of ectoparasiticides; environmental and lifestyle conditions; and travel history.

The only selection criteria for the clinics were to be small animal or mixed practices located in the north of Spain, and to accept to collaborate in the study.

Whole blood was obtained for each dog in EDTA tubes between January 2017 and December 2018 to evaluate the seroprevalence of the different vector-borne pathogens assessed in this study.

Two commercial rapid diagnostic tests, the 4DX SNAP® Test (IDEXX Laboratories, Westbrook, ME, USA) and the *Leishmania* SNAP*®* Test (IDEXX Laboratories) were used for *D. immitis* antigen-detection, and *E. canis*, *B. burgdorferi*, *Anaplasma* spp. and *L. infantum* antibody detection, in blood samples according to the manufacturer’s instructions. A previous study reported sensitivity and specificity, respectively, using the 4Dx SNAP® Test for antigen detection of *D. immitis* (99.2%, 100%) and antibody detection of *E. canis* (96.2%, 100%), *B. burgdorferi* (98.8%, 100%) and *A. phagocytophilum* (99.1%, 100%) [14]. On the other hand, the sensitivity and specificity for the *Leishmania* SNAP® Test were 96.3% and 99.2%, respectively, as per the manufacturer’s instructions (IDEXX Laboratories).

### Statistical analysis

Statistical associations between the results obtained from the different serological tests and the epidemiological and clinical data were assessed using Chi-square test or Fisher’s exact test for categorical variables, where appropriate. Confidence interval (95% CI) values were also calculated. Bonferroni-correction for *P*-values was applied for seroprevalence pairwise comparisons. Odds ratios (OR) were also obtained to assess different risk factors. Student’s t-test was used to compare means of numerical data. The significance level was established at *P* < 0.05. Statistical analysis was carried out using the software SAS, version 9.4 (SAS Institute, Cary, NC, USA).

## Results

Epidemiological data are shown in Table 1. A total of 63 out of the 556 dogs (11.33 ± 1.3%) were seropositive to any vector-borne pathogen tested. The overall seroprevalence for each pathogen was as follows: 8.99 ± 1.2% (50/556) for *L. infantum*; 1.26 ± 0.5% (7/556) for *Anaplasma* spp.; 0.9 ± 0.4% (5/556) for *E. canis*; 0.72 ± 0.4% (4/556) for *B. burgdorferi*, and 0.18 ± 0.2% (1/556) for *D. immitis.* Detailed distribution of seropositivity to the different pathogens by region is provided in Fig. 1. Only 3 dogs (0.54 ± 0.3%) were seropositive to two different pathogens (*L. infantum* + *E. canis*, *L. infantum + Anaplasma* spp. and *E. canis + Anaplasma* spp.). There was no statistically significant association between seropositivity to more than one agent and the different evaluated variables.

**Table 1:** Comparison of vector-borne pathogens prevalence in samples of dogs from the north of Spain in relation to the epidemiological data collected

Epidemiological data	Total no. of dogs (%)	Number of positive dogs (%)					
		*L. infantum*	*D. immitis*	*E. canis*	*Anaplasma* spp.	*B. burgdorferi*	Any seropositive
Number of dogs	556	50 (8.99)	1 (0.18)	5 (0.90)	7 (1.26)	4 (0.72)	63 (11.33)
Prevalence (%) (95% CI)		8.99 ± 1.21 (6.61–11.37)	0.18 ± 0.18 (0–0.53)	0.90 ± 0.40 (0.11–1.69)	1.26 ± 0.47 (0.33–2.19)	0.72 ± 0.36 (0.02–1.42)	11.33 ± 1.34 (8.70–13.96)
Clinical signs suggestive of vector-borne diseases	556						
Yes	283 (50.90)	43 (15.19)*	1 (0.35)	5 (1.77)	3 (1.06)	2 (0.71)	51 (18.02)*
No	273 (49.10)	7 (2.56)	0 (0)	0 (0)	4 (1.47)	2 (0.73)	12 (4.40)
Season of sample collection	545						
Spring	158 (28.99)	15 (9.49)	0 (0)	0 (0)	1 (0.63)	0 (0)	16 (10.13)
Summer	118 (21.65)	7 (5.93)	0 (0)	4 (3.39)*	1 (0.85)	1 (0.85)	11 (9.32)
Autumn	155 (28.44)	14 (9.03)	0 (0)	1 (0.65)	1 (0.65)	0 (0)	15 (9.68)
Winter	114 (20.92)	11 (9.65)	1 (0.88)	0 (0)	4 (3.51)	2 (1.75)	17 (14.91)
Age	543						
Puppy (< 1–year-old)	64 (11.79)	1 (1.56)	0 (0)	0 (0)	1 (1.56)	0 (0)	2 (3.13)
Young (1–5 years-old)	234 (43.09)	19 (8.12)	1 (0.43)	1 (0.43)	3 (1.28)	3 (1.28)	25 (10.68)
Adult (5–10-years-old)	187 (34.44)	17 (9.09)	0 (0)	3 (1.60)	3 (1.60)	1 (0.53)	23 (12.30)
Old (> 10 years-old)	58 (10.68)	10 (17.24)*	0 (0)	1 (1.72)	0 (0)	0 (0)	10 (17.24)
Breed	554						
Yes	397 (71.66)	38 (9.57)	1 (0.25)	3 (0.76)	4 (1.01)	1 (0.25)	44 (11.08)
No	157 (28.34)	12 (7.64)	0 (0)	2 (1.27)	3 (1.91)	3 (1.91)	19 (12.10)
Sex	556						
Male	315 (56.65)	32 (10.16)	1 (0.32)	2 (0.63)	5 (1.59)	3 (0.95)	41 (13.02)
Female	241 (43.35)	18 (7.47)	0 (0)	3 (1.24)	2 (0.83)	1 (0.41)	22 (9.13)
Weight	548						
Small (< 10 kg)	104 (18.98)	6 (5.77)	0 (0)	0 (0)	0 (0)	2 (1.92)	8 (7.69)
Small-medium (10–20 kg)	170 (31.02)	19 (11.18)	0 (0)	4 (2.35)	2 (1.18)	1 (0.59)	25 (14.71)
Medium-large (> 20–30 kg)	173 (31.57)	18 (10.40)	0 (0)	0 (0)	4 (2.31)	1 (0.58)	21 (12.14)
Large (> 30 kg)	101 (18.43)	6 (5.94)	1 (0.99)	1 (0.99)	1 (0.99)	0 (0)	8 (7.92)
Ticks infestation	556						
Yes	64 (11.51)	5 (7.81)	0 (0)	0 (0)	2 (3.13)	1 (1.56)	8 (12.5)
No	492 (88.49)	45 (9.15)	1 (1.2)	5 (1.02)	5 (1.02)	3 (0.61)	55 (11.18)
Use of ectoparasiticides	556						
Yes	362 (65.11)	33 (9.12)	0 (0)	4 (1.10)	4 (1.10)	1 (0.28)	39 (10.77)
No	194 (34.89)	17 (8.76)	1 (0.52)	1 (0.52)	3 (1.55)	3 (1.55)	24 (12.37)
Living outdoor/indoor	545						
Indoor	219 (40.18)	13 (5.94)	0 (0)	4 (1.83)	1 (0.46)	0 (0)	17 (7.76)
Outdoor	164 (30.09)	23 (14.02)*	1 (0.61)	0 (0)	2 (1.22)	2 (1.22)	19 (11.63)
Mixed	162 (29.72)	14 (8.68)	0 (0)	1 (0.62)	4 (2.47)	2 (1.23)	27 (16.46)*
Living area	544						
Urban	203 (37.32)	13 (6.4)	0 (0)	4 (1.97)	0 (0)	0 (0)	16 (7.88)
Periurban	120 (22.06)	11 (9.17)	0 (0)	0 (0)	1 (0.83)	0 (0)	12 (10)
Rural	221 (40.63)	26 (11.76)	1 (0.45)	1 (0.45)	6 (2.71)*	4 (1.81)*	35 (15.84)*
National travel history	556						
Yes	165 (29.68)	12 (7.27)	0 (0)	0 (0)	4 (2.42)	0 (0)	16 (9.7)
No	391 (70.32)	38 (9.72)	1 (0.26)	5 (1.28)	3 (0.77)	4 (1.02)	47 (12.02)
International travel history	556						
Yes	42 (7.57)	3 (7.14)	0 (0)	0 (0)	1 (2.38)	0 (0)	4 (9.52)
No	514 (92.43)	47 (9.16)	1 (0.19)	5 (0.97)	6 (1.17)	4 (0.78)	59 (11.5)
Clinical signs suggestive of:	283						
Anaplasmosis	71 (12.77)	2 (2.82)	0 (0)	1 (1.41)	3 (4.23)*	1 (1.41)	6 (8.45)
Ehrlichiosis	126 (22.66)	7 (5.56)	0 (0)	3 (2.38)	1 (0.79)	1 (0.79)	10 (7.94)
Dirofilariosis	33 (5.94)	2 (6.06)	0 (0)	0 (0)	0 (0)	0 (0)	2 (6.06)
Leishmaniasis	127 (22.84)	32 (25.20)*	0 (0)	1 (0.79)	1 (0.79)	1 (0.79)	33 (25.98)*
Borreliosis	50 (8.99)	0 (0)	0 (0)	0 (0)	0 (0)	1 (2)	1 (2)
Autonomous communities	556						
Aragon	57 (10.25)	14 (24.56)^a,b,c,d^	0 (0)	1 (1.75)	1 (1.75)	0 (0)	15 (26.32)^h,i^
Asturias	79 (14.21)	1 (1.27)^a,e^	0 (0)	1 (1.27)	2 (2.53)	2 (2.53)	6 (7.59)
Basque Country	80 (14.39)	4 (5)^b^	0 (0)	1 (1.25)	2 (2.5)	0 (0)	6 (7.50)
Cantabria	91 (16.37)	3 (3.3)^c,f^	0 (0)	0 (0)	1 (1.10)	0 (0)	4 (4.40)^h,j^
Catalonia	97 (17.45)	13 (13.40)	0 (0)	1 (1.03)	0 (0)	2 (2.06)	15 (15.46)
Galicia	80 (14.39)	2 (2.50)^d,g^	0 (0)	0 (0)	0 (0)	0 (0)	2 (2.50)^i,k^
Navarra	72 (12.95)	13 (18.06)^e,f,g^	1 (1.39)	1 (1.39)	0 (0)	0 (0)	15 (20.83)^j,k^

^a–k^Groups with the same lowercase letter show statistically significant differences between them, when analyzing pairwise comparisons

* *P* ≤ 0.05

Clinical signs were found in more than half of dogs included in the study, 328 (58.99%) out of 556 dogs (Table 2). Specifically, clinical signs suggestive of these vector-borne diseases were found in 283/556 (50.9%) dogs. Among them, 18.02 ± 2.3% (*n* = 51) were seropositive to any pathogen tested. Individually, 15.19 ± 2.1% (*n* = 43) were seropositive to *L. infantum*, 1.77 ± 0.8% (*n* = 5) to *E. canis*, 1.06 ± 0.4% (*n* = 3) to *Anaplasma* spp., 0.71 ± 0.6% (*n* = 2) to *B. burgdorferi*, and 0.35 ± 0.4% (*n* = 1) to *D. immitis*. These rates were significantly higher in the subset of dogs with clinical signs in the case of *L. infantum* (OR: 6.8, 95% CI: 3.00–15.42, *P* < 0.0001), and in the case of seropositivity for any vector-borne pathogen tested (OR: 4.78, 95% CI: 2.49–9.19, *P* < 0.0001).

**Table 2:** Presence of clinical signs in relation with serological antibody detection of *Leishmania infantum*, *Ehrlichia canis*, *Borrelia burgdorferi*, *Anaplasma* spp., and antigen-detection of *Dirofilaria immitis*

Clinical sign	No. of dogs (*N* = 556)	*L. infantum* (*N* = 50)	*D. immitis* (*N* = 1)	*E. canis* (*N* = 5)	*Anaplasma* spp. (*N* = 7)	*B. burgdorferi* (*N* = 4)	Any seropositive (*N* = 63)
	*n* (%)	*n* (%)	*n* (%)	*n* (%)	*n* (%)	*n* (%)	*n* (%)
Total	328 (58.99)	41 (12.50)*	1 (0.30)	5 (1.52)	4 (1.22)	4 (1.22)	52 (15.85)*
Apathy	192 (34.53)	18 (9.38)	1 (0.52)	5 (2.60)*	3 (1.56)	1 (0.52)	25 (13.02)
Weakness	155 (27.88)	15 (9.68)	1 (0.65)	4 (2.58)*	1 (0.65)	1 (0.65)	21 (13.55)
Anorexia	117 (21.04)	12 (10.26)	1 (0.85)	5 (4.27)*	2 (1.71)	1 (0.85)	19 (16.24)*
Weight loss	98 (17.63)	12 (12.24)	0 (0)	3 (3.06)*	2 (2.04)	1 (1.02)	17 (17.35)*
Lethargy	97 (17.45)	8 (8.25)	0 (0)	2 (2.06)	1 (1.03)	1 (1.03)	11 (11.34)
Anemia	95 (17.09)	14 (14.74)*	0 (0)	3 (3.16)*	2 (2.11)	1 (1.05)	18 (18.95)*
Fever	90 (16.19)	3 (3.33)*	0 (0)	3 (3.33)*	1 (1.11)	0 (0)	6 (6.67)
Exercise intolerance	76 (13.67)	9 (11.84)	0 (0)	0 (0)	0 (0)	1 (1.32)	10 (13.16)
Dermatological signs	73 (13.13)	17 (23.29)*	0 (0)	0 (0)	0 (0)	1 (1.37)	18 (24.66)*
Orthopedic signs	59 (10.61)	9 (15.25)	0 (0)	1 (1.69)	0 (0)	0 (0)	9 (15.25)
Lymphadenomegaly	52 (9.35)	19 (36.54)*	0 (0)	2 (3.85)	0 (0)	0 (0)	20 (38.46)*
Digestive signs	52 (9.35)	6 (11.54)	1 (1.92)	3 (5.77)*	0 (0)	0 (0)	8 (15.38)
Muscular atrophy	37 (6.65)	12 (32.43)*	0 (0)	0 (0)	0 (0)	0 (0)	12 (32.43)*
Hemorrhagic signs	33 (5.94)	5 (15.15)	0 (0)	1 (3.03)	1 (3.03)	0 (0)	7 (21.21)
Ocular signs	31 (5.58)	8 (25.81)*	0 (0)	1 (3.23)	1 (3.23)	0 (0)	8 (25.81)*
Renal disease	27 (4.86)	6 (22.22)*	0 (0)	1 (3.70)	0 (0)	0 (0)	6 (22.22)
Neurological signs	26 (4.68)	2 (7.69)	0 (0)	0 (0)	0 (0)	0 (0)	2 (7.69)
Splenomegaly	20 (3.60)	1 (5.00)	0 (0)	1 (5.00)	1 (5)	0 (0)	3 (15.00)
Cough	17 (3.06)	1 (5.88)	0 (0)	0 (0)	0 (0)	0 (0)	1 (5.88)
Tachycardia	17 (3.06)	1 (5.88)	0 (0)	0 (0)	1 (5.88)	0 (0)	2 (11.76)
Dyspnea/tachypnea	17 (3.06)	2 (14.29)	0 (0)	1 (5.88)	1 (5.88)	0 (0)	3 (21.43)
Hepatomegaly	10 (1.80)	0 (0)	0 (0)	1 (10.00)	0 (0)	0 (0)	1 (10.00)
Cranial vena cava syndrome	1 (0.18)	0 (0)	0 (0)	0 (0)	0 (0)	0 (0)	0 (0)

* *P* ≤ 0.05

Considering the group without suggestive clinical signs of the same diseases (49.1%, *n* = 273), the seroprevalence rates were 4.4 ± 1.2% (*n* = 12) for any pathogen tested. When it was evaluated individually these rates were 2.56 ± 1% (*n* = 7) for *L. infantum*, 1.47 ± 0,7% (*n* = 4) for *Anaplasma* spp., and 0.73 ± 0.5% (*n* = 2) for *B. burgdorferi*.

When evaluating the statistical association between presence of ticks and detection of antibodies against different tick vector-borne agents no significant associations were detected: *Anaplasma* spp. (OR: 3.14, 95% CI: 0.60–6.54, *P* = 0.18); *E. canis* (OR: 1.01, 95% CI: 1.00–1.02, *P* = 1); and *B. burgdorferi* (OR: 2.59, 95% CI: 0.26–25.25, *P* = 0.39). However, the use of ectoparasiticide treatment was statistically associated with the absence of ticks during physical examination of the dogs (*χ*^2^ = 27.09, *df* = 1, *P* < 0.001).

*Leishmania infantum* was the most prevalent pathogen and the only one detected in all the autonomous communities included in the present study. The highest seroprevalence (24.56 ± 5.7%) in northern Spain was detected in Aragon. In fact, *L. infantum* was significantly more prevalent in this community than in all the other communities included in the study, except for Navarra (18.06 ± 4.5%) and Catalonia (13.4 ± 3.5%). Asturias presented the lowest seroprevalence for *L. infantum* (1.27 ± 1.3%) (Fig. 1; Table 1). Dogs seropositive to *L. infantum* without travel history, or with travel only to other northern regions of the country, were detected in all the regions included in the study. Dogs seropositive for *L. infantum* were older (6.97 ± 3.80 years-old) than seronegative dogs (5.37 ± 3.64 years-old) (*t*_(541)_ = 2.88, *P* = 0.0042). Specifically, seropositivity for *L. infantum* was higher in dogs older than 10 years (OR: 2.38, 95% CI: 1.12–5.07, *P* = 0.0203).

Dogs living outdoors (*n* = 23, 14.02%) were significantly associated with *L. infantum* antibody detection (OR: 2.13, 95% CI: 1.18–3.85, *P* = 0.0101). Fifty-two per cent (26/50) of the positive dogs were living in rural areas (OR: 1.66, 95% CI: 0.93–2.98, *P* = 0.08).

A total of 32 out of 50 seropositive dogs had suggestive clinical signs of canine leishmaniasis (OR: 7.69, 95% CI: 4.14–14.28, *P* < 0.0001). Significant associations were detected between *L. infantum* seropositivity and anemia (OR: 2.04, 95% CI: 1.05–3.95, *P* = 0.0316), fever (OR: 0.31, 95% CI: 0.09–1.01, *P* = 0.04), dermatological signs (OR: 4.14, 95% CI: 2.17–7.91, *P* < 0.0001), lymphadenomegaly (OR: 8.78, 95% CI: 4.49–17.19, *P* < 0.0001), muscular atrophy (OR: 6.07, 95% CI: 2.83–13.03, *P* < 0.0001), ocular alterations (OR: 4.00, 95% CI: 1.69–9.49, *P* = 0.0038), and renal disease (OR: 3.15, 95% CI: 1.21–8.21, *P* = 0.0266).

The overall prevalence for *D. immitis* was 0.18 ± 0.2% (Fig. 1). *Dirofilaria immitis* antigen was detected only in a 3-year-old male German shepherd from Navarra, without travel history (1.39 ± 1.34%). The clinical signs were apathy and jaundice, and the dog was also positive in a previous test for leptospirosis. It was a guard dog that lived in a farm outside and without prophylactic treatments against arthropods.

Antibodies against *E. canis* were detected in 5 out of 556 dogs (0.9 ± 0.4%). Aragon was the region with the highest prevalence (1.75 ± 1.7%). All dogs from Cantabria and Galicia included in this study were negative (Fig. 1). None of the seropositive dogs had ever travelled out of their region of origin.

Four of the five (80%) canine blood samples positive to *E. canis* were collected during summer (OR: 14.95, 95% CI: 1.65–135, *P* = 0.0088).

All the dogs positive to *E. canis* belonged to the subset of dogs with suggestive signs of vector-borne diseases (OR: 10.8, 95% CI: 0.59–196.3, *P* = 0.06), and three of them had clinical signs clearly suggestive of ehrlichiosis (OR: 5.22, 95% CI: 0.86–31.59, *P* = 0.0795).

Regarding clinical signs, significant associations were detected between *E. canis* antibody detection and apathy (OR: 21.38, 95% CI: 1.18–388.80, *P* = 0.0047), weakness (OR: 10.6, 95% CI: 1.17–95.56, *P* = 0.023), anorexia (OR: 42.97, 95% CI: 2.36–782.92, *P* = 0.0004), weight loss (OR: 7.2, 95% CI: 1.19–43.68, *P* = 0.046), anemia (OR: 7.48, 95% CI: 1.23–45.42, *P* = 0.0373), fever (OR: 8, 95% CI: 1.32–48.58, *P* = 0.0321), and gastrointestinal signs (OR: 15.37, 95% CI: 2.51–94.19, *P* = 0.0068).

Seropositivity to *Anaplasma* spp. was 1.26 ± 0.5% (*n* = 7), Asturias being the area with highest prevalence rate (2.53 ± 1.8%). *Anaplasma* spp. were not detected in any dog included in the study from Galicia, Navarra and Catalonia (Fig. 1, Table 1). Five out of seven samples were collected in colder months, but no statistically significant differences were found (OR: 2.6, 95% CI: 0.50–13.49, *P* = 0.2802). Finally, 6 out of the 7 seropositive dogs were living in rural areas (OR: 8.99, 95% CI: 1.07–75.17, *P* = 0.0199). A total of 3 out of 7 presented clearly suggestive clinical signs of anaplasmosis (OR: 5.3, 95% CI: 1.16–24.22, *P* = 0.0478).

Antibodies against *B. burgdorferi* were only detected in Asturias (2.53 ± 1.8%, *n* = 2), and Catalonia (2.06 ± 1.4%, *n* = 2). None of the positive dogs had previously travelled out of their area of origin. The four dogs seropositive for *B. burgdorferi* were living in a rural area (OR: 13.39, 95% CI: 0.71–249.89, *P* = 0.0268).

When considering all the dogs seropositive to any evaluated vector-borne pathogen in this study, the prevalence rate was 11.33 ± 1.3% (*n* = 63). A total of 51 out of 63 dogs had clinical signs compatible with vector-borne diseases (OR: 4.78, 95% CI: 2.49–9.19, *P* < 0.0001) and 35 out of 63 were living in a rural area (OR: 1.98, 95% CI: 1.17–3.37, *P* = 0.0103).

Fifty-two out of these 63 dogs presented some clinical signs (OR: 3.72, 95% CI: 1.89–7.29, *P* < 0.0001). Specifically, there was a significant relationship between seropositivity to any pathogen of the study and anorexia (OR:1.74, 95% CI: 0.97–3.11, *P* = 0.05), weigh loss (OR: 1.88, 95% CI: 1.03–3.44, *P* = 0.0384), anemia (OR: 2.16, 95% CI: 1.19–3.93, *P* = 0.01), dermatological signs (OR: 3.18, 95% CI: 1.72–5.89, *P* = 0.0001), lymphadenomegaly (OR: 6.7, 95% CI: 3.53–12.71, *P* < 0.0001), muscular atrophy (OR: 4.4, 95% CI: 2.09–9.29, *P* < 0.0003), and ocular alterations (OR: 2.97, 95% CI: 1.27–6.96, *P* = 0.0165).

With regard to age, the group of dogs seropositive to any vector-borne pathogen was older (6.70 ± 3.62 years-old) than the seronegative group (5.46 ± 3.66 years-old) (*t*_(541)_ = 2.69, *P* = 0.0074). In this sense, the puppy group (2/63, 3.17%) had lower positivity to vector-borne pathogens when compared with the remaining age groups (61/63, 96.82%) (OR: 0.22, 95% CI: 0.05–0.96, *P* = 0.0277).

## Discussion

To the best of our knowledge, this is the first multicenter survey performed assessing different canine vector-borne pathogens in all the autonomous communities of northern Spain, and a total of 11.33 ± 1.3% of dogs were seropositive. Previous studies on other vector-borne pathogens have been performed in this area of Spain, focusing particularly on *Babesia* spp. and *L. infantum* [3, 21–27]. Therefore, this study emerges from the need to provide support to the veterinary practitioners working within the different regions of northern Spain, by identifying potential vector-borne pathogens, clinical signs and risk factors to be considered.

The study shows different prevalence rates in the seven autonomous communities of northern Spain (Fig. 1). These differences could be based on climatic conditions (temperature and humidity), the associated presence of the vectors involved in the life-cycle of the pathogens, and the relationship between dogs and their respective vectors considering a wide range of risk factors [6, 9].

Irrespective of the differences observed in prevalence, a remarkable finding is the presence of autochthonous cases in all the northern regions of Spain, taking into account the detection of seropositive dogs to the evaluated vector-borne pathogens without travel history or without travel history out of the regions included in the study.

The prevalence of *L. infantum* was found to be considerably variable depending on the area of study (from 24.56 ± 5.7% in northern Aragon to 1.27 ± 1.3% in Asturias). In fact, when comparing the autonomous communities, Aragon, Navarra and Catalonia, were the regions with the highest seroprevalence rates of *L. infantum*, probably due to the adequate environment that allows sand fly development and completion of the biological life-cycle of the parasite [4].

The north of Spain, especially the central and northwestern regions, has been an area classically considered as non-endemic of canine leishmaniasis [3, 25, 26]. In this respect, previous studies carried out using IFAT in shelter dogs from north Spain (Cantabria, Asturias, Galicia and the Basque Country) found a total seroprevalence of 3% for *L. infantum* in the Cantabrian coast [25]. *Leishmania infantum* antibodies were found in all the regions included in this study. The prevalence rates found in Catalonia and Galicia are in concordance with those obtained in a previous multicenter study performed in different areas of Spain [9]. Similarly, the rates found in Catalonia and Pyrenean areas are similar to those found in a recent study in the same area [28]. Furthermore, a study in the Basque Country detected a high prevalence using PCR for *L. infantum* DNA (28%, 44/156) among wild carnivores, strongly suggesting the presence of the parasite in this area [10]. However, caution is recommended when comparing studies due to potential differences in the diagnostic techniques used, sample size, animal species evaluated, and the origin of the dogs, among other factors [9, 10].

The results presented here reinforce the hypothesis about the global spread of *L. infantum* in the north of Spain, including more areas than previously considered [3, 26, 29]. This highlights the importance of including *L. infantum* in the differential diagnosis in areas traditionally considered as non-endemic or peri-endemic for this pathogen, especially in Aragon and Navarra. In this respect, a previous retrospective study of visceral and cutaneous human leishmaniasis prevalence, showed a low rate in northern Spain (0–0.5 cases/100,000 inhabitants) in comparison with other central or southern regions. However, the rate in Navarra (0.5–2.0/100,000 inhabitants) and especially the rates in Aragon and Catalonia (2–3.5/100,000 inhabitants), were considerable higher [11].

The higher seropositivity for *L. infantum* found in dogs older than 10 years-old could be explained by longer exposure to sand flies over time, and/or immune depletion described in the course of infection, especially affecting elderly dogs. Similar to this study, a bimodal trend has been previously described in canine leishmaniasis [4, 25]. Although some studies showed the predisposition of certain breeds or sex to canine leishmaniasis [30, 31], there was no statistical association in the present study.

Living outdoors represents a risk factor probably due to the increased contact with the vectors (*Phlebotomus perniciosus* or *P. ariasi*), which has been detected in previous studies in northern Spain, including some areas previously considered free of leishmaniasis [12, 26, 29].

Canine leishmaniasis is a chameleonic disease that shows up with many possible clinical signs [8], all caused by either the direct action of the parasite or the deposition of circulating immune complexes. Nevertheless, due to the high number of dogs with clinical signs included in this study, clinical signs associated to leishmaniasis were suspected and recognized by most of the practitioners working in the areas of study. It could be explained by the fact that canine leishmaniasis is well known among practitioners despite this wide diversity of clinical signs [9].

*Ehrlichia canis* seropositivity was also detected in all the evaluated areas, except for Galicia and Cantabria. However, *E. canis* has previously been detected in the southern area of Galicia [21] and in northern Portugal [15]. The absence of *E. canis* seropositivity in Galicia and antibodies detected from only one dog from Asturias agrees with a previous study carried out in dogs from the northwest of Spain (Galicia and Asturias), where all 75 dogs tested (IFAT) were seronegative to *E. canis* [23]. On the other hand, a previous study showed considerably higher seroprevalence for *E. canis* in Catalonia (*n* = 5/49; 10.2% in Barcelona) [32]. The overall seroprevalence rate of ehrlichiosis detected in our study (0.9 ± 0.4%) was lower than in previous studies in the same area where *E. canis* was reported to be the most prevalent rickettsial agent in Spain, with a seroprevalence of 5% [9].

The significant detection of *E. canis* antibodies during the summer months agrees with the peak of activity found in the area for *R. sanguineus* from March to July [6] and the subsequent incubation period of the infection (1–3 weeks) [17]. The higher seroprevalence found in dogs living indoors in our study also matches with the higher presence of *R. sanguineus* detected in indoor-living dogs previously described in Spain [6]. This result could be explained by the endophilic behavior of *R. sanguineus*, living in anthroponotic areas, in close contact with dogs and humans [33, 34]. Furthermore, ticks were not detected in any dog positive to *E. canis* at the time of clinical examination, in concordance with the efficacy of ectoparasiticide treatments reported herein. This fact could also be explained for the *R. sanguineus* host detachment in the different stages of the life-cycle [34, 35], or detection of antibodies that may persist for several months or years reported for *E. canis*, and also for *Anaplasma* spp. [17].

Apathy, weakness, anorexia, weight loss, anemia, fever and gastrointestinal signs were significantly associated with *E. canis* seropositivity. All these clinical signs have been classically described in dogs with *E. canis* infection [17, 36]. On the other hand, four out of the five dogs positive to *E. canis* were theoretically protected using ectoparasiticide prevention treatments. This fact could reflect incorrect use of these treatments or an insufficient pattern in their use, highlighting the importance not only of use, but also of correct administration guidelines to prevent this and others tick-borne infections [17].

*Anaplasma phagocytophilum* and *A. platys* could not be differentiated with the diagnostic technique used in this study due to test cross-reactivity [14]. Thus, this technique allowed us to detect exposure to the genus level only. Differentiation of these species requires a PCR assay or, with lower sensitivity, blood smear visualization [17]. Starting from this limitation, *Anaplasma* spp. were the most seroprevalent tick-borne pathogens detected in the study, with Asturias being the area with the highest seroprevalence rate. This prevalence can be expected considering the abundance of the potential vectors (*I. ricinus* and *R. sanguineus*) in the area [6]. In contrast, a previous serological study did not find dogs with antibodies against *Anaplasma* spp. in Asturias [9]. Five out of the seven samples positive for *Anaplasma* spp. were collected in colder months, although without statistically significant association. Even when the association between seropositivity against *Anaplasma* spp. and the presence of clinical signs of anaplasmosis could support the possibility of infection in these dogs [17], these results should be cautiously interpreted, taking into account that seropositivity could only reflect exposure to the agent.

Antibodies against *B. burgdorferi* were only detected in dogs from Asturias and Catalonia, with the overall seroprevalence in northern areas (0.72%) similar to those found in a previous study performed in Spain (0.4%), where there was a similar distribution in the different regions included [9]. In this sense, a similar low seroprevalence rate (0.66%, 3/460) was detected in a previous study of dogs from Mallorca and Catalonia (Barcelona and Tarragona) using the same test [32]. Our findings are in contrast with a previous study performed in southern Galicia (Pontevedra and Ourense) where the seroprevalence by IFAT for *B. burgdorferi* was 6.26% (30/479) in owned dogs [21]. Differences in the technique and in the studied population could explain the variability in the seroprevalence found in different studies.

*Ixodes* spp. are the main vectors of borreliosis in Europe and are present in the area of study without clear seasonality for either *I. ricinus* or *I. hexagonus* [6], probably explaining the lack of association in our study with the season of sample collection. The detection of *B. burgdorferi* antibodies in dogs living in rural areas in this study was in concordance with the presence of *Ixodes* spp. on dogs living in rural areas in these regions of Spain [6]. Contact with vectors and/or potential sylvatic reservoirs could be higher in dogs living in this environment. In this sense, *B. burgdorferi* antibodies have also been previously detected in wild canids (wolves and foxes) from northern Spain [5]. The lack of association between suspected clinical signs of borreliosis and the detection of antibodies against *B. burgdorferi* reported herein has been previously shown in northern Spain [37].

The clinical picture in the only dog from Navarra in which *D. immitis* antigen was detected should be treated with caution, due to the co-infection with *Leptospira* spp., because the latter could justify most of clinical signs described in this dog.

All dogs with co-infections were seropositive to *Anaplasma* spp., *E. canis* or *L. infantum*, suggesting an immunocompromised status that could facilitate co-infection as previously described [38, 39], or shared vectors and/or routes of transmission. In this sense, *E. canis* and *Anaplasma* spp. are commonly found co-infecting dogs [17].

Finally, when considering all the dogs seropositive to any vector-borne pathogen, this study showed a great variation of prevalence among regions, from 26.32 ± 5.8% in northern Aragon to 2.5 ± 1.7% in Galicia. These variations could be due to differences in the environment, vector abundance and/or the interactions between dogs and vectors [6]. Globally, this study showed that living in a rural area represents a risk factor, probably due to the higher exposure to arthropods and wildlife reservoirs of the vector-borne pathogens previously reported in these areas [5, 10]. Similarly, dogs younger than one year-old seem less likely to be infected, probably due to their limited exposure to pathogens; however, they will have an increased opportunity for exposure over time [17, 40, 41].

Real seroprevalence rates could probably be higher than detected herein considering that dogs included in the study were owned dogs, and most of them were protected against ectoparasites. This is in line with the higher previously reported rates in kennel dogs [25], and wild mammals [5] in northern Spain.

## Conclusions

To the best of our knowledge, this is the first serological multicenter study focused on selected vector-borne pathogens performed in owned dogs from northern Spain, covering all regions from northeast to northwest. The present study confirms that vector-borne pathogens are prevalent and autochthonous in the areas studied, showing high prevalence rates especially in Navarra, northern Aragon and northern Catalonia. The results presented here highlight the significance of the veterinary control of vector-borne pathogens, and the implementation of effective prophylactic measures against arthropods in the northern areas of Spain. The expansion of canine vector-borne diseases to non-endemic areas and the wide variety of clinical signs presented by the affected dogs is a challenge for practitioners. Surveillance programmes must be established in order to have a better knowledge, management and control of the canine vector-borne pathogens. However, further molecular studies evaluating different pathogens in dogs from these areas are warranted to better understand the epidemiological and clinical situation.

## Data Availability

All data generated or analyzed during this study are included in this published article.

## Abbreviations

Ab/s: antibody/ies
Ag/s: antigen/s
CI: confidence interval
OR: odds ratio
